# A decade of receptor discordance and phenotypic transitions in metastatic breast cancer: a single-center retrospective study of 363 cases

**DOI:** 10.3389/fonc.2026.1797175

**Published:** 2026-04-20

**Authors:** Sijie Chen, Qianqian Sun, Li Yan, Jiao Yang, Jin Yang

**Affiliations:** 1 Cancer Center, the First Affiliated Hospital of Xi’an Jiaotong University, Xi’an, China; 2 Precision Medicine Center, the First Affiliated Hospital of Xi’an Jiaotong University, Xi’an, China; 3 Department of Medical Oncology, the First Affiliated Hospital of Xi’an Jiaotong University, Xi’an, China

**Keywords:** biomolecular status, dynamic receptor assessment, metastatic breast cancer, prognosis, receptor heterogeneity

## Abstract

**Purpose:**

To investigate real-world changes in the expression of estrogen receptor (ER), progesterone receptor (PR), human epidermal growth factor receptor 2 (HER2), and corresponding molecular subtypes in primary and metastatic breast cancer, as well as their influencing factors and impact on prognosis.

**Methods:**

This retrospective study analyzed 436 paired lesion samples of primary and metastatic breast cancer treated between 2013 and 2023. Statistical evaluation was conducted to examine receptor discordance (ER, PR, HER2) and clinicopathological correlations.

**Result:**

Receptor discordance rates between primary and metastatic lesions were 25.1% for ER, 33.3% for PR, 32.8% for HER2, and 33.8% for molecular subtypes. PR showed the highest discordance, frequently transitioning from positive to negative. ER discordance predominantly involved the loss of low expression. HER2 discordance was highest in liver metastases (59.3%). Molecular subtype changes were most common in HR+/HER2- tumors (42.6%). ER discordance correlated with neoadjuvant chemotherapy and endocrine therapy, while HER2 discordance was linked to targeted therapy and lung metastases. Loss of hormone receptor (HR) or HER2 expression was associated with poorer overall survival (OS) (HR: median OS 95 vs. 70 months, P = 0.0018; HER2: median OS 78 vs. 68 months, P = 0.025). Conversely, sustained or acquired HR/HER2 positivity improved OS.

**Conclusions:**

Heterogeneity in ER, PR, HER2, and molecular subtype expression significantly influences prognosis and treatment outcomes in metastatic breast cancer. Reassessment of receptor status in metastatic lesions is essential for optimizing individualized treatment strategies and improving patient outcomes.

## 1 Introduction

Representing 32% of cancers in women, breast cancer is the most frequently diagnosed malignancy and the second major contributor to cancer-related deaths. Approximately 20% of early-stage breast cancer cases will eventually progress to metastatic disease, presenting a significant threat to women’s health (1, 2). Advances in early detection and treatment have significantly improved breast cancer management; however, distant metastasis remains a crucial clinical issue. The recurrence risk ranges from 10% to 41%, determined by pathological traits and lymphatic spread (3, 4).

Estrogen receptor (ER), progesterone receptor (PR), and human epidermal growth factor receptor 2 (HER2) are critical biomarkers in breast cancer, playing pivotal roles in determining prognosis and guiding therapeutic strategies. The analysis of biomarkers in primary and metastatic sites is fundamental for determining treatment plans and prognostic predictions. Molecular subtypes correlate with unique prognoses, metastasis tendencies, and individualized therapeutic pathways (5).

Studies have highlighted significant discrepancies in receptor expression between primary and metastatic lesions, including ER (14.0%-21.1%), PR (31.1%-40.3%), and HER2 (7.8%-15.0%) (6–10). Increasing evidence points to substantial disparities in receptor expression between primary breast cancer and distant metastatic sites (6, 11–13). This variation extends to the genomic and transcriptomic levels (14). In light of this, clinical guidelines from NCCN, ESMO, EGTM, and ASCO unanimously recommend the reassessment of hormone receptors in metastatic lesions when feasible (8, 15–18). The heightened focus on tailored care and personalized treatment in breast cancer management highlights the necessity of studying potential biological alterations during the disease course (19).

This research focuses on assessing the prevalence of receptor discordance observed between primary breast tumors and corresponding metastatic sites. Our secondary aim is to investigate the determinants of receptor inconsistencies. Furthermore, we evaluate the prognostic impact of receptor discordance on clinical results.

## 2 Material and methods

### 2.1 Study design and sample

A retrospective review was conducted on 363 cases of recurrent/metastatic breast cancer treated at the First Affiliated Hospital of Xi’an Jiaotong University over a 10-year period (2013–2023), focusing on clinical and pathological characteristics. This study relied on the hospital’s electronic medical record system and pathology department’s diagnostic data to include patients with complete ER, PR, and HER2 status results for both primary breast cancer lesions and their corresponding metastatic sites, determined through immunohistochemistry (IHC) and *in situ* hybridization (ISH). Patients were excluded if they had incomplete IHC or ISH data for either primary or metastatic lesions, were male, had a second malignancy, or had incomplete follow-up information.

The analytical unit was predefined according to study endpoints. Receptor discordance analyses were performed at the lesion level (436 paired primary–metastatic lesion samples). Survival analyses (DFS and OS) were conducted at the patient level (363 patients).

For patients with multiple metastatic biopsies, lesion-level analyses included all available paired samples to describe biological heterogeneity. Sensitivity analyses restricted to the first metastatic biopsy per patient were additionally performed to assess robustness.

Ethical approval for this research was granted by the Ethics Committee of the First Affiliated Hospital of Xi’an Jiaotong University, reference number: No.XJTU1AF2022LSK-312.

### 2.2 ER, PR, and HER2 assessment

Retrospective data collection included ER, PR, and HER2 receptor status, histopathological features, and clinical information from medical records. Receptor status determination was performed as per the most updated standards outlined by the American Society of Clinical Oncology (ASCO) and the College of American Pathologists (CAP) at that time (20). HER2 was regarded as positive when the IHC score was 3+, or 2+ accompanied by HER2 amplification confirmed through ISH testing. HER2 status was considered negative for an IHC score of 0, 1+, or 2+ with ISH demonstrating the absence of amplification. For HER2-negative tumors, low HER2 expression was specified by an IHC score of 1+ or 2+ with a negative ISH outcome, while HER2-zero was defined by an IHC score of 0. ER and PR were deemed positive when at least 1% of tumor cells demonstrated IHC staining. Low ER expression was described as ER positivity within 1-10% of tumor cells (12). Hormone receptor (HR) positivity was classified if the tumor exhibited either ER or PR positivity. The immunohistochemical positivity of ER and PR was characterized by the presence of yellow or brown granules within the nuclei of tumor cells. Conversely, HER2 expression was defined by distinct brown membranous staining exhibiting a characteristic complete or incomplete ‘chicken-wire’ pattern.

### 2.3 Adjuvant therapy and follow-up

The follow-up period commenced from the date of the initial surgery and continued until the detection of metastasis, which included both local and distant recurrence. Local recurrence was characterized by metastatic spread to the ipsilateral breast, chest wall, or associated regional lymph nodes. In contrast, distant metastasis encompassed dissemination to the contralateral breast, chest wall, lymphatic structures (contralateral and bilateral supraclavicular lymph nodes), bones, and visceral organs. Follow-up strategies integrated outpatient consultations, inpatient evaluations, and regular telephone follow-ups.

Disease-free survival (DFS) was characterized as the time span from the surgical excision of primary breast cancer to the confirmation of recurrence or metastatic progression. Overall survival (OS) was measured from the point of recurrence or metastasis diagnosis to the time of death.

### 2.4 Statistical methods

Descriptive methods were applied to characterize clinicopathological data from patients with primary and metastatic breast cancer, presenting results as median values, case numbers, and proportions. Kappa statistics were employed to determine concordance in ER, PR, and HER2 status between primary and metastatic sites. To explore factors associated with hormone receptor (HR) and HER2 discordance, qualitative analyses relied on the chi-square test or Fisher’s exact test, and univariate approaches were applied. Multivariate analysis utilized a Cox proportional hazards model to investigate phenotypic discordance. Kaplan-Meier survival curves were employed to visualize survival distributions, with group differences assessed using the log-rank test. Multivariate regression analysis was conducted through the Cox proportional hazards model, calculating hazard ratios (HRs) and 95% confidence intervals (CIs) to evaluate risks.

Statistical computations and data visualization utilized SPSS 25.0, Python 3.9, and R 4.4.1. All tests were performed as two-tailed analyses, with statistical significance defined by a P-value of less than 0.05.

## 3 Result

### 3.1 Patient characteristics and follow-up results

**Figure 1:**
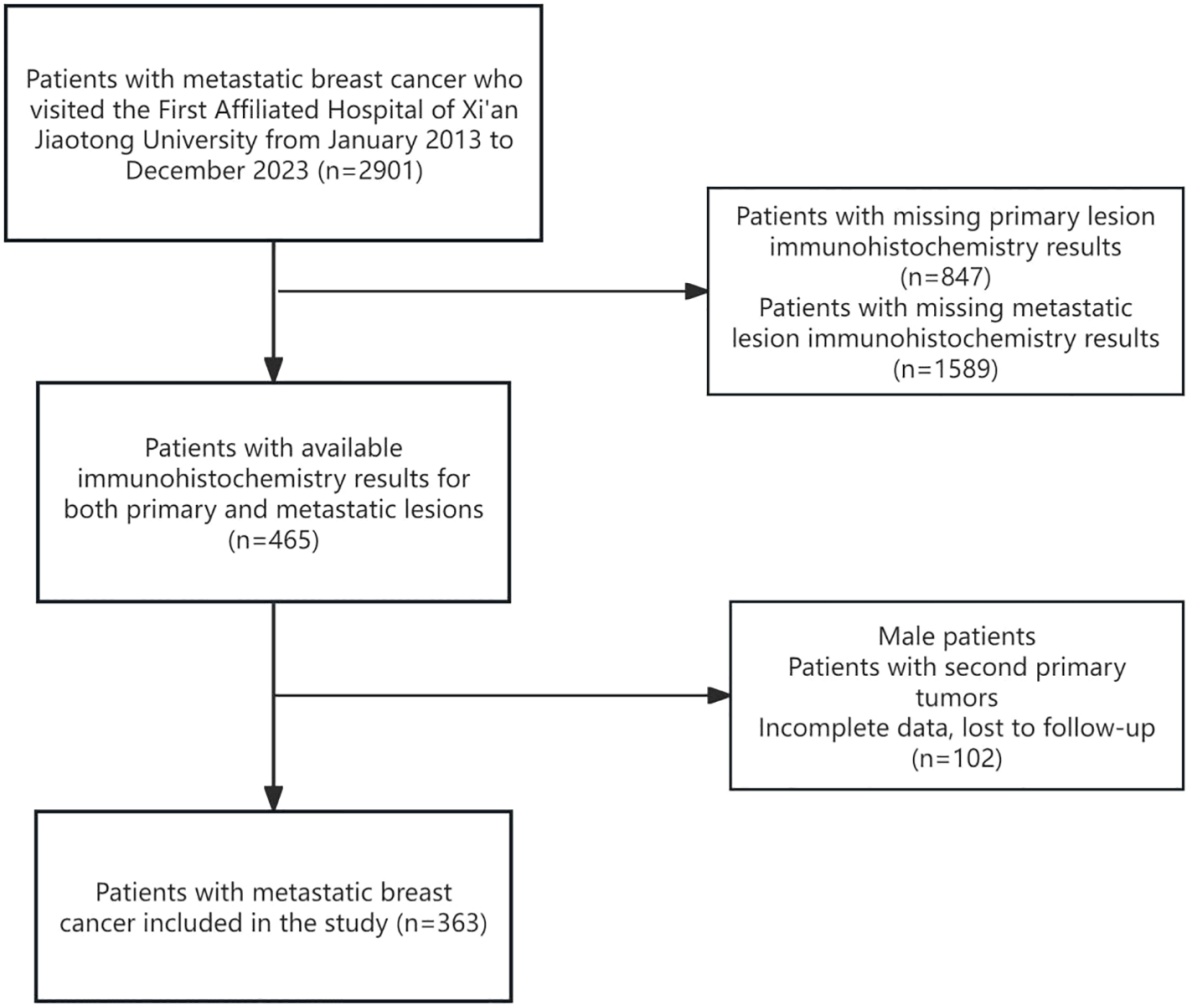
Flowchart of the study. 363 patients were selected from 2901 patients with metastatic breast cancer in the past 10 years according to the inclusion and exclusion criteria.

A retrospective review of 363 patients with breast cancer and confirmed metastatic lesions was conducted at the First Affiliated Hospital of Xi’an Jiaotong University between January 2013 and December 2023. Exclusion criteria applied to 2,358 patients, including 2,436 without ER, PR, or HER2 results for either primary or metastatic lesions, 102 male patients, those with secondary tumors, and individuals lost to follow-up. Finally, 363 patients and 436 lesions meeting the criteria were included in the analysis (**Figure 1**).

### 3.2 Clinicopathological features

This study collected immunohistochemical data for ER, PR, and HER2 from 436 matched lesion pairs in 363 metastatic breast cancer patients. Among them, 61 had two to four distant metastatic sites, and 31 patients (7.1%) presented with distant metastases at their initial diagnosis.

The demographic and clinicopathological characteristics of the patients revealed a median age of 49.3 years. HR+/HER2- was the predominant primary lesion subtype, representing 42.0%. The median time from initial pathological diagnosis to the appearance of metastases was 33.6 months. At presentation, 7.1% of patients were diagnosed with stage IV disease. Disease-free survival (DFS) was ≤24 months for 51.8% of the cases, whereas 41.1% had DFS periods exceeding 24 months.

Regarding treatment before re-biopsy of the metastatic lesions, 20.4% of patients had received neoadjuvant chemotherapy, 92.7% had undergone adjuvant chemotherapy, 58.5% had received targeted therapy, 58.3% had been treated with endocrine therapy, and 68.1% had undergone radiotherapy. Sixty-one patients had biopsies from two or more metastatic lesions, resulting in a total of 134 pairs of matched lesions, representing approximately 16.8% of the cohort. Approximately 22% of patients experience distant metastases in the liver, making it the most common biopsy site.

### 3.3 Heterogeneity of receptor expression in primary and metastatic lesions

The overall inconsistency rate for ER status between primary and metastatic lesions was 25.1% (109/436). Specifically, 2.8% (12/436) of patients exhibited a change of ER from positive to low expression, 6.9% (30/436) from positive to negative, 1.4% (6/436) from low expression to positive, 7.3% (32/436) from low expression to negative, while 3.4% (15/436) from negative to positive, and 3.2% (14/436) from negative to low expression. The Kappa test indicated moderate consistency in ER expression between primary and metastatic lesions (Kappa = 0.619). Bowker’s Test of Symmetry showed statistically significant changes in ER status between primary and metastatic lesions (P = 0.002).

PR status showed an overall inconsistency rate of 33.3% (145/436) between primary and metastatic lesions. Of these, 24.8% (108/436) transitioned from positive to negative, and 8.5% (37/436) transitioned from negative to positive. The Kappa consistency test showed a moderate level of agreement in PR expression between primary and metastatic lesions (Kappa value = 0.322). The paired χ2 test confirmed that changes in PR status were statistically significant (*P* < 0.001).

The overall inconsistency rate for HER2 status between primary and metastatic lesions was 32.8% (143/436). Changes included 6.9% (30/436) from positive to low expression, 2.8% (12/436) from positive to negative, 2.5% (11/436) from low expression to positive, 11.0% (48/436) from low expression to negative, 1.6% (7/436) from negative to positive, and 8.0% (35/436) from negative to low expression. The Kappa consistency test indicated a moderate level of agreement in HER2 expression between primary and metastatic lesions (Kappa value = 0.500). Bowker’s Test of Symmetry showed that changes in HER2 status were statistically significant (*P* = 0.007). Detailed data are presented in **Tables 1** and **2**.

**Table 1:** Descriptive analysis and Kappa concordance test determined the expression of ER, PR, HER2 receptor status and molecular subtypes in primary and metastatic lesions.

Receptors	Receptor status	Primary lesion	Metastatic lesions	Kappa
ER	positive	138	117	0.619
	low-expression	130	118	
	negative	168	201	
PR	positive	207	136	0.322
	negative	229	300	
HER2	positive	196	172	0.500
	low-expression	130	136	
	negative	110	128	
Subtype	HR+/HER2+	107	133	0.523
	HR+ /HER2-	183	118	
	HR- /HER2+	89	120	
	HR- /HER2-	57	65	

**Table 2:** Descriptive analysis of changes in ER, PR, and HER2 receptor status n (%).

Receptors	Changes in receptor status	Numbers	Total n(%)
ER	Positive→Low	12	109 (25.1%)
	Positive→Negative	30	
	Low→Positive	6	
	Low→Negative	32	
	Negative→Positive	15	
	Negative→Low	14	
PR	Positive→Negative	108	145 (33.3%)
	Negative→Positive	37	
HER2	Positive→Low	30	143 (32.8%)
	Positive→Negative	12	
	Low→Positive	11	
	Low→Negative	48	
	Negative→Positive	7	
	Negative→Low	35	

**Table 3** summarizes the alterations in molecular subtypes observed between primary breast cancer lesions and their corresponding metastatic counterparts. Metastatic lesions exhibited an increase in the HR+/HER2+ subtype from 24.5% to 30.5%, while the incidence of triple-negative subtypes slightly increased from 13.1% to 14.9%. Among HR+/HER2- patients, 14.2% (26/183) transitioned to the triple-negative subtype, and 28.4% (52/183) shifted to the HER2+ subtype. For triple-negative patients, 7.0% (4/57) transitioned to the HR+/HER2- subtype, and 33.3% (19/57) shifted to the HER2+ subtype. The highest inconsistency rate in molecular subtypes was observed in HR+/HER2- patients, with only 57.4% (105/183) maintaining the original subtype in metastatic lesions. Conversely, HR-/HER2+ patients exhibited the lowest inconsistency rate, with 78.7% (70/89) retaining the original subtype in metastatic lesions.

**Table 3:** Descriptive analysis of molecular subtypes of primary and metastatic lesions n (%).

Molecular subtypes	Change of status	Numbers	Total n(%)
HR+ HER2+ (n=107)	→HR+ HER2-	7	33 (30.8%)
	→HR- HER2+	24	
	→HR- HER2-	2	
HR+ HER2- (n=183)	→HR+ HER2+	40	78 (42.6%)
	→HR- HER2+	12	
	→HR- HER2-	26	
HR- HER2+ (n=89)	→HR+ HER2+	14	19 (21.3%)
	→HR+ HER2-	2	
	→HR- HER2-	3	
HR- HER2- (n=57)	→HR+ HER2+	5	23 (40.4%)
	→HR+ HER2-	4	
	→HR- HER2+	14	

**Figure 2:**
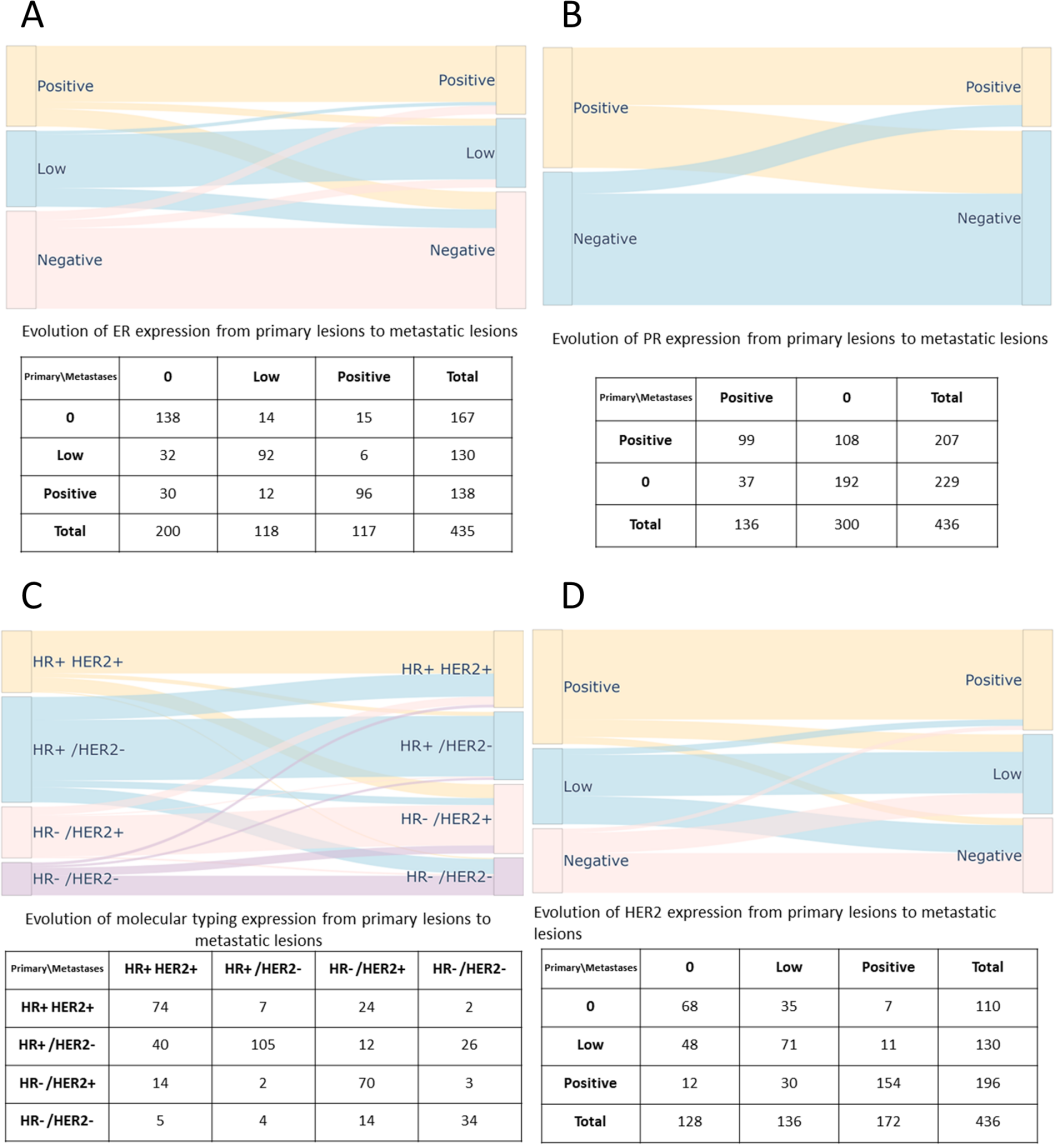
Sankey diagrams of changes in ER, PR, HER2 status and molecular subtypes between primary breast cancer and metastases. **(A)** Changes in ER status between primary and metastatic lesions. **(B)** Changes in PR status between primary and metastatic lesions. **(C)** Changes in molecular subtypes between primary and metastatic lesions. **(D)** Changes in HER2 status between primary and metastatic lesions.

Sankey diagrams were applied to illustrate the dynamics of ER, PR, and HER2 expression, along with changes in molecular subtypes, between primary and metastatic lesions (**Figure 2**). Among these biomarkers, PR displayed the highest rate of discordance, with a considerable number of cases exhibiting a shift from PR-positive to PR-negative status. The most prominent alteration in ER expression was a transition from low levels to a negative status, a pattern similarly observed for HER2 expression. Regarding molecular subtypes, the most frequent transformation was from HR+/HER2- to HR+/HER2+.

**Figure 3:**
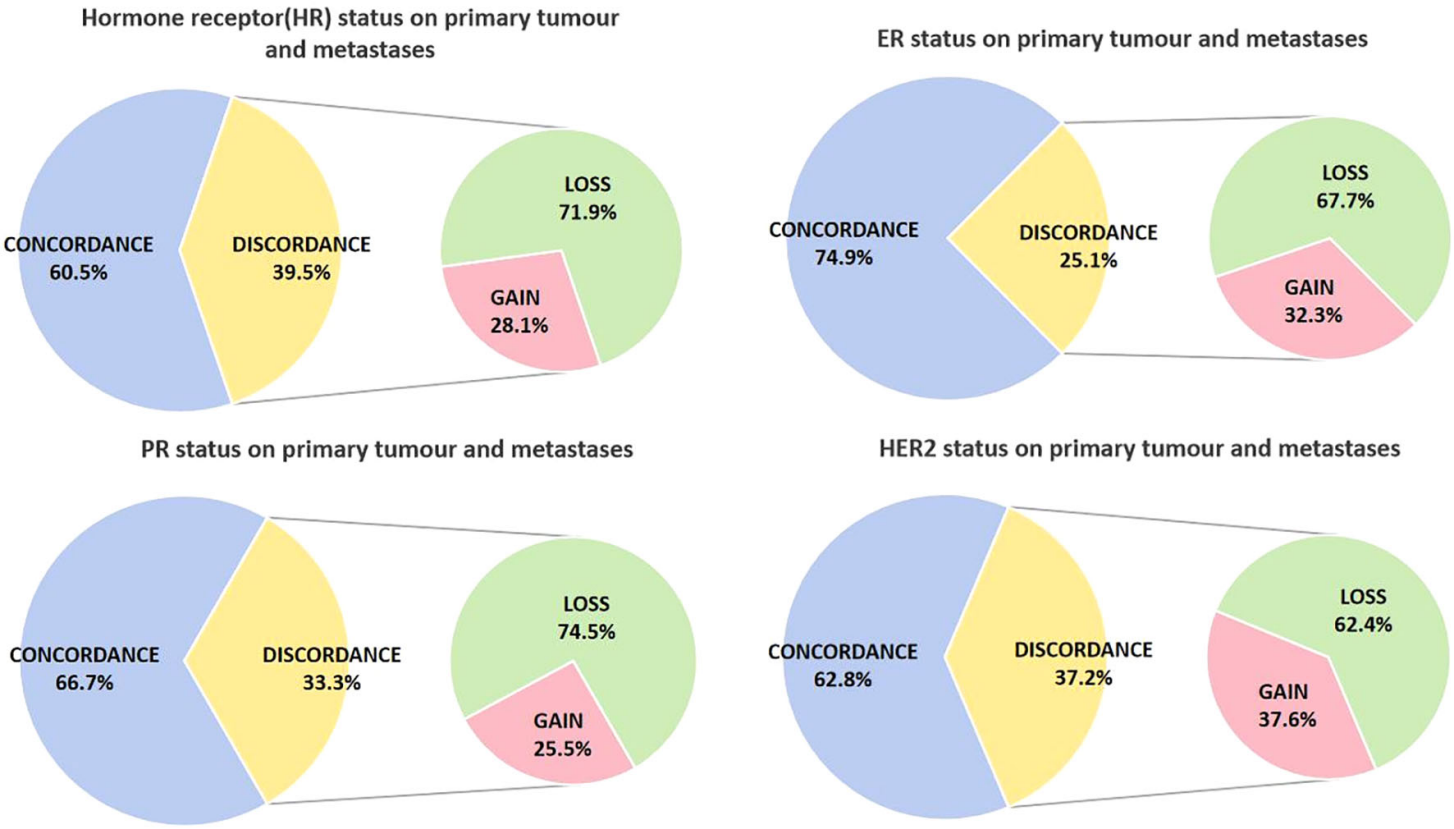
Modification of ER, PR, and HER2 status between primary tumor and metastasis. **(A)** Changes in ER status between primary and metastatic lesions. **(B)** Changes in PR status between primary and metastatic lesions. **(C)** Changes in molecular subtypes between primary and metastatic lesions. **(D)** Changes in HER2 status between primary and metastatic lesions.

At the point of metastatic diagnosis, the overall discordance in HR status between primary and metastatic tumors was 39.5%, characterized by a loss of expression in 71.9% of cases and a gain in 28.1%. Regarding ER status, 25.1% of cases exhibited alterations, with 67.7% showing a loss of expression and 32.3% demonstrating a gain. For PR status, the modification rate reached 33.3%, with 74.5% experiencing expression loss and 25.5% showing expression gain. Finally, HER2 status displayed a discordance rate of 37.2%, with 62.4% of cases showing a reduction in expression and 37.6% exhibiting an increase (**Figure 3**).

**Figure 4:**
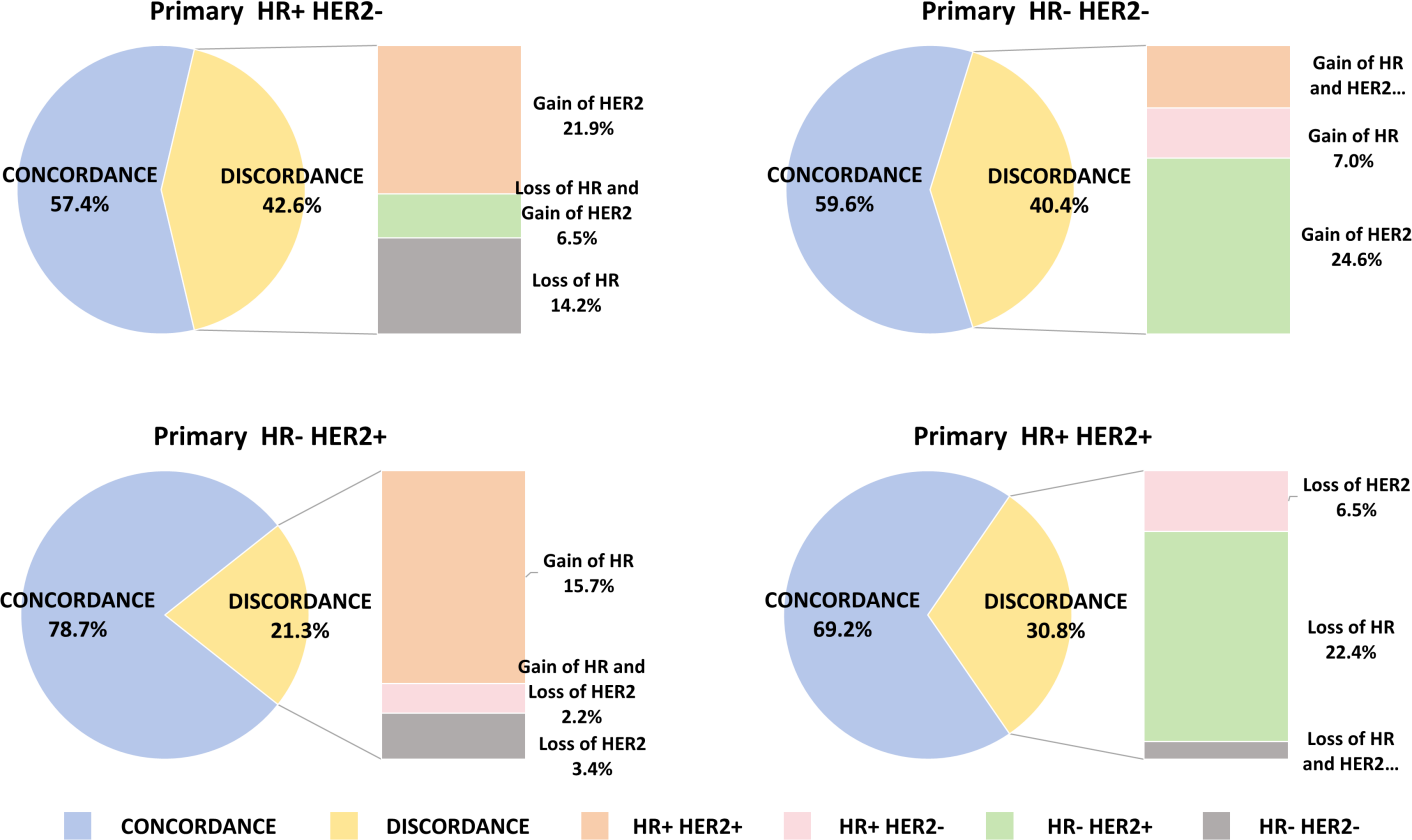
Breast cancer subtypes on primary tumor and metastatic disease. **(A)** the primary molecular classification is HR+HER2- and the acquisition of HER2 is the main feature in metastatic lesions. **(B)** The primary molecular classification is HR-HER2- and the acquisition of HER2 is the main feature in metastatic lesions. **(C)** The primary molecular classification is HR-HER2+. In metastatic lesions, the main feature is the acquisition of HR. **(D)** The primary molecular classification is HR+HER2+. The main feature of the metastatic lesion is the loss of HR.

Among phenotypic subtypes, primary HR+/HER2- tumors exhibited the highest rate of changes, at 42.6%. Specifically, 14.2% of these tumors showed loss of HR expression, 21.9% experienced loss of HER2 expression, and 6.5% showed loss of both HR and HER2. In contrast, primary HR-/HER2+ tumors displayed phenotypic changes in 21.3% of cases, with a majority demonstrating HR gain (73.7%). Detailed data are presented in **Figure 4**.

**Figure 5:**
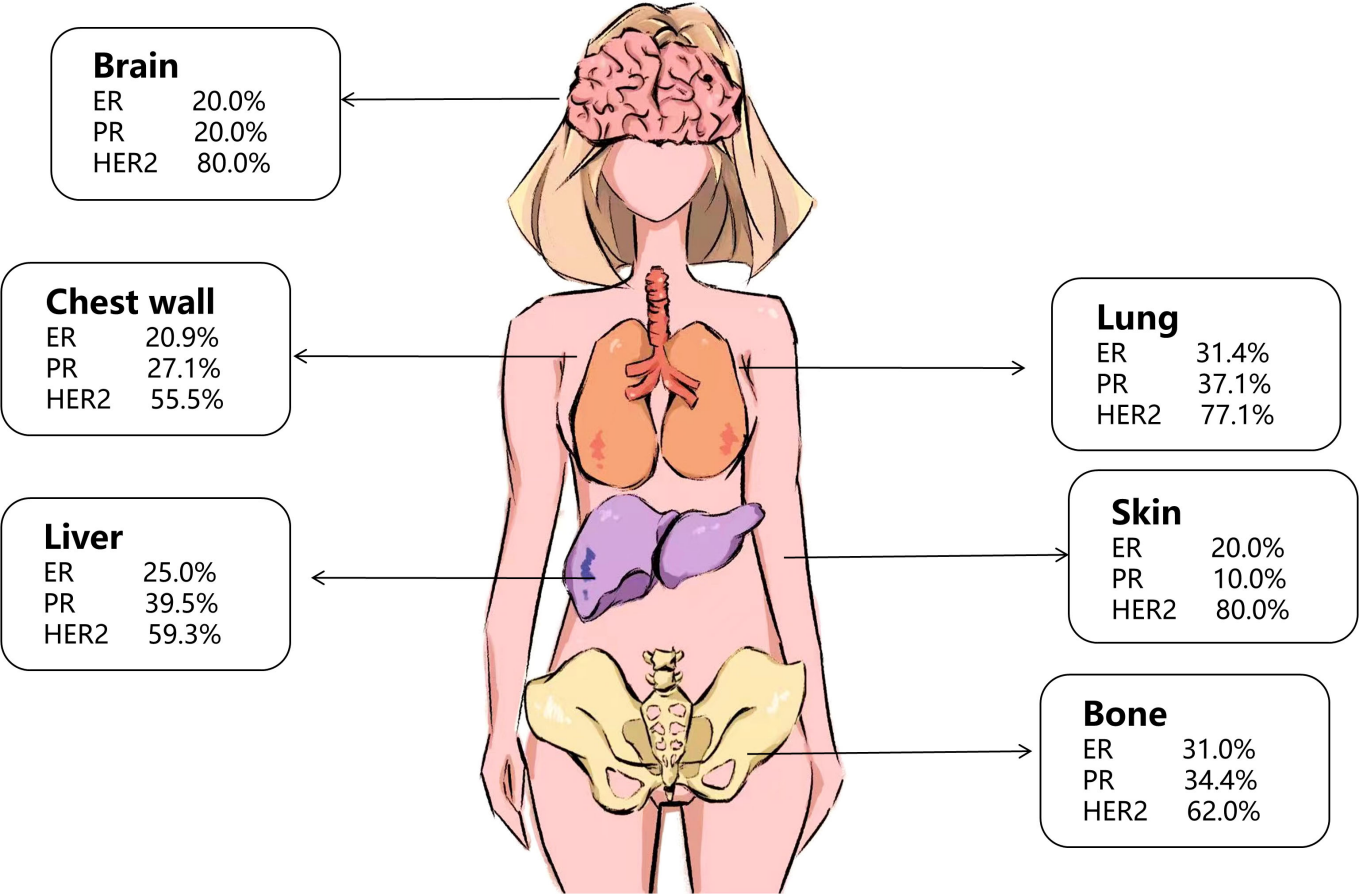
Inconsistency of ER, PR, and HER2 between primary lesions and metastatic lesions in different metastatic organs.

Receptor heterogeneity is also evident between different metastatic lesions. The rate of HER2 status change is highest in liver metastases (59.3%), and similar trends are observed in lung metastases, bone metastases, and chest wall metastases. Detailed data are presented in **Figure 5** and **Supplementary Table 1**.

A total of 61 patients underwent biopsies of multiple metastatic lesions, ranging from 2 to 4 lesions per patient, resulting in 134 pairs of matched lesions (**Table 4**). The discordance rates were 35% (47/134) for ER status, 30.5% (41/134) for PR status, and 60.4% (81/134) for HER2 status.

**Table 4:** Inconsistency of receptor status between different metastatic sites in the same patient.

Receptors	Changes in receptor status	Numbers	Total n(%)
ER(n=134)	Positive→Low	12	47 (35.0%)
	Positive→Negative	30	
	Low→Positive	6	
	Low→Negative	32	
	Negative→Positive	15	
	Negative→Low	14	
PR(n=134)	Positive→Negative	108	41 (30.5%)
	Negative→Positive	37	
HER2(n=134)	Positive→Low	30	81 (60.4%)
	Positive→Negative	12	
	Low→Positive	11	
	Low→Negative	48	
	Negative→Positive	7	
	Negative→Low	35	

### 3.4 Factors associated with receptor conversion

Univariate analysis was performed (**Table 5**) to identify potential determinants of receptor conversion in metastatic lesions. For ER inconsistency, significant factors included neoadjuvant chemotherapy (P = 0.002), endocrine therapy (P < 0.001), and the number of biopsied metastatic lesions (P = 0.002). PR inconsistency was significantly associated with endocrine therapy (P < 0.001) and disease-free survival (DFS) (P = 0.002). Additionally, the analysis highlighted targeted therapy (P < 0.001), endocrine therapy (P = 0.033), and lung metastasis (P = 0.007) as potential contributors to HER2 conversion.

**Table 5:** Univariate analysis of variables associated with discordant receptor status.

Factors		ER			PR			HER2	
	Concordant	Discordant	P value	Concordant	Discordant	P value	Concordant	Discordant	P value
Age at first diagnosis			0.864			0.660			0.170
<50	154	53		136	71		70	137	
≥50	172	57		155	74		92	137	
TNM stage at first diagnosis			0.226			0.088			0.081
I-III	300	105		266	139		155	250	
IV	26	5		25	6		7	24	
Menopausal status			0.800			0.650			0.259
Yes	111	36		96	51		60	87	
No	215	74		195	94		102	187	
Family history of cancer			0.193			0.385			0.132
Yes	69	17		54	32		38	48	
No	257	93		237	113		124	226	
Neoadjuvant therapy			0.002			0.246			0.450
Yes	78	11		64	25		30	59	
No	248	99		227	120		132	215	
Adjuvant chemotherapy			0.226			0.088			0.081
Yes	300	105		266	139		155	250	
No	26	5		25	6		7	24	
Targeted therapy			0.954			0.007			<0.001
Anti-HER2 targeted therapy	132	43		129	46		26	149	
CDK4/6 inhibitors	60	20		43	37		44	36	
No	134	47		119	62		92	89	
Endocrine therapy			<0.001			<0.001			0.033
Yes	169	85		137	117		105	149	
No	157	25		154	28		57	125	
Radiation therapy			0.151			0.960			0.255
Yes	216	81		198	99		105	192	
No	110	29		93	46		57	82	
Number of biopsied metastases			0.002			0.432			0.490
1 site	239	63		198	104		109	193	
≥2 sites	87	47		93	41		53	81	
DFS			0.345			0.002			0.207
≤24	148	47		143	52		73	122	
>24	152	58		123	87		82	128	
Stage IV at initial diagnosis	26	5		25	6		7	24	
Liver metastasis			0.953			0.136			0.426
Yes	72	24		58	38		39	57	
No	254	86		233	107		123	217	
Lung metastasis			0.192			0.451			0.007
Yes	48	22		44	26		16	54	
No	278	88		247	119		146	220	
Bone metastasis			0.456			0.885			0.929
Yes	20	9		19	10		11	18	
No	306	101		272	135		151	256	
Chest wall metastasis			0.330			0.197			0.133
Yes	93	85		59	22		36	45	
No	157	64		232	123		126	229	

A multivariate analysis was conducted using the Cox proportional hazards model (**Table 6**). The results identified tumor stage as a significant predictor of survival, with higher stages correlating with a notably reduced risk of death (p = 0.015, HR = 0.3715, 95% CI: 0.1667–0.8280). Targeted therapy was also associated with a significantly lower mortality risk (p = 0.0072, HR = 0.7645, 95% CI: 0.6284–0.9300). While lung metastasis approached statistical significance (p = 0.057, HR = 0.65), it did not meet the threshold, suggesting a potential but inconclusive effect on survival. Other variables, including menopausal status, family cancer history, endocrine therapy, radiotherapy, liver metastasis, bone metastasis, and chest wall involvement, showed p-values > 0.05, indicating no statistically significant impact on survival. The model demonstrated a concordance index of 0.708, reflecting a predictive accuracy of 70.8%. Highly significant p-values (p < 0.001) for the Likelihood Ratio, Wald, and Score (Log-rank) tests confirmed the robust fit of the model.

**Table 6:** Cox proportional hazards model analysis of variables related to receptor status discordance.

Factors	HR	95% CI	P value
Age at first diagnosis	1.069	0.7459 - 1.5306	0.7177
TNM stage at first	0.3715	0.1667 - 0.8280	0.0154
Menopausal status	0.9126	0.6278 - 1.3265	0.6317
Family history of cancer	0.8769	0.6156 - 1.2490	0.4665
Neoadjuvant therapy	2.121	1.5147 - 2.9696	< 0.001
Adjuvant chemotherapy	4.643e+05	0.0000 - Inf	0.9930
Targeted therapy	0.7645	0.6284 - 0.9300	0.0072
Endocrine therapy	0.9014	0.6679 - 1.2166	0.4974
Radiation therapy	0.9163	0.6777 - 1.2387	0.5696
Number of biopsied metastases	0.5396	0.3919 - 0.7429	0.0001
DFS	0.3718	0.2774 - 0.4984	< 0.001
Liver metastasis	0.8853	0.6094 - 1.2861	0.5226
Lung metastasis	0.6529	0.4204 - 1.0138	0.0576
Bone metastasis	0.5833	0.3020 - 1.1265	0.1084
Chest wall metastasis	1.023	0.7137 - 1.4660	0.9020

### 3.5 Impact of receptor conversion on DFS and OS

The patient survival follow-up in this study concluded on December 31, 2023, with a median follow-up time of 88 months (95% CI: 78–96 months).

**Figure 6:**
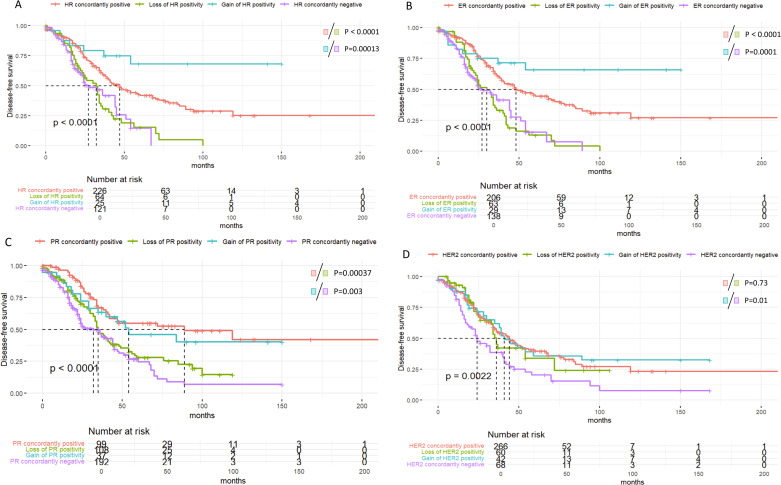
Kaplan-Meier survival curves illustrating disease-free survival (DFS) stratified by receptor status changes over time in breast cancer patients. **(A)** Disease-free survival (DFS) based on Hormone Receptor (HR) status changes, showing significant differences between HR subgroups (p < 0.0001). **(B)** DFS stratified by Estrogen Receptor (ER) status changes, highlighting survival differences between ER subgroups (p < 0.0001). **(C)** DFS for different Progesterone Receptor (PR) status groups, with significant p-values indicating survival differences among PR subgroups (p < 0.0001). **(D)** DFS in HER2 status groups, showing survival differences between HER2-positive and HER2-negative subgroups (p = 0.0022).

In terms of disease-free survival (DFS), patients with consistently positive HR status demonstrated the most favorable prognosis, highlighting the potential association between persistent HR positivity and improved outcomes. In contrast, those with HR status transitioning from positive to negative exhibited the steepest decline in survival, indicating that HR loss may serve as a predictor of disease progression (P < 0.0001) (**Figure 6**). The maintenance or restoration of ER and PR positivity was correlated with improved prognosis. Similarly, HER2 positivity exerted a beneficial effect on survival, with patients transitioning from HER2-negative to HER2-positive achieving significantly better outcomes than those remaining HER2-negative (P = 0.01).

**Figure 7:**
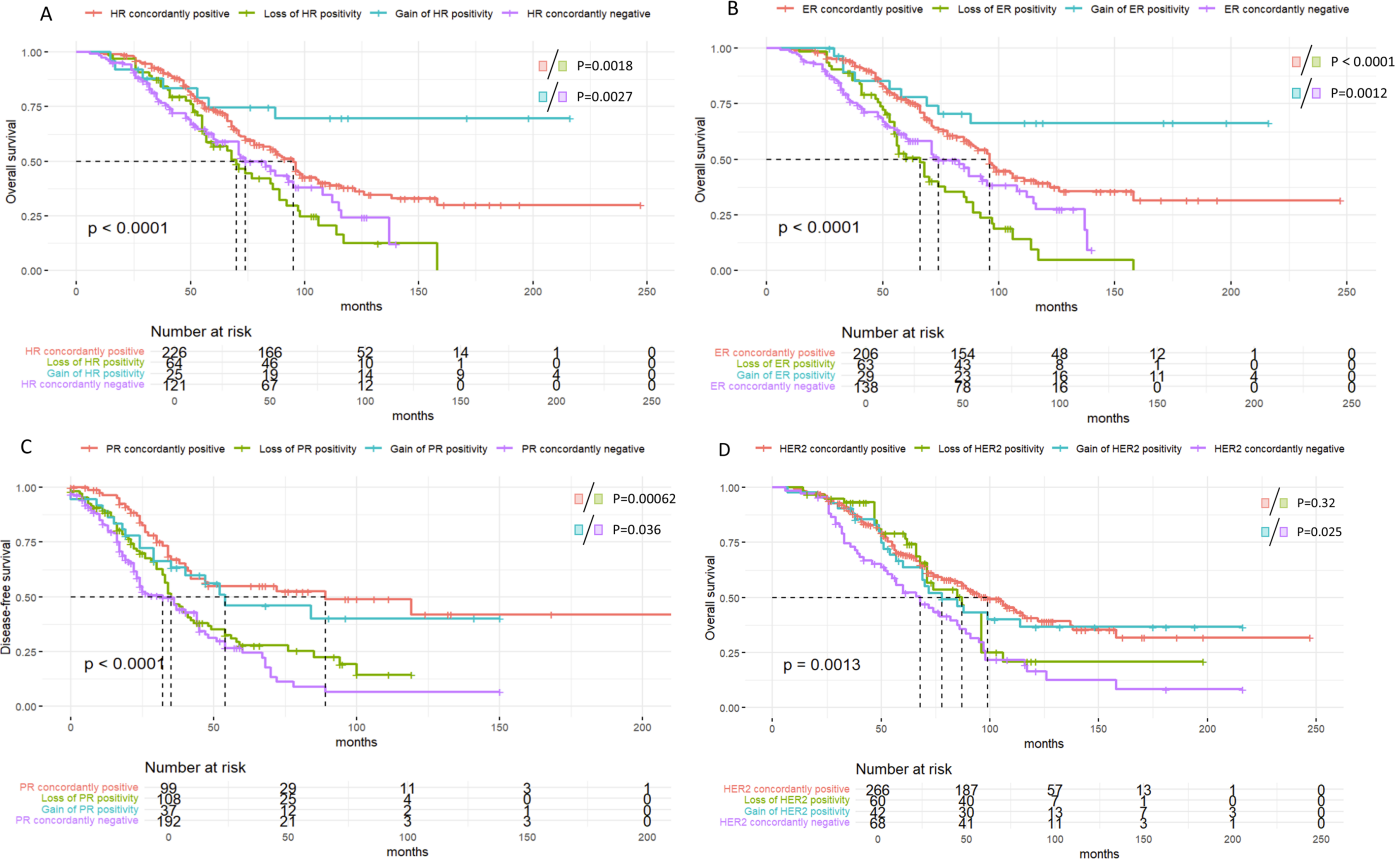
Kaplan-Meier survival curves illustrating overall survival (OS) based on receptor status changes over time in breast cancer patients. **(A)** Overall survival (OS) by Hormone Receptor (HR) status changes, with significant survival differences across HR subgroups (p < 0.0001). **(B)** OS stratified by Estrogen Receptor (ER) status changes, showing distinct survival outcomes among ER subgroups (p < 0.0001). **(C)** OS based on Progesterone Receptor (PR) status changes, with significant survival differences observed (p < 0.00062). **(D)** OS by HER2 status changes, highlighting differences in survival across HER2 subgroups (p = 0.0013).

Overall survival (OS) exhibited trends similar to those of DFS. The loss of HR or HER2 positivity significantly correlated with poorer OS outcomes (HR: median OS of 95 vs. 70 months, P = 0.0018; HER2: median OS of 78 vs. 68 months, P = 0.025). In contrast, the maintenance or acquisition of HR and HER2 positivity was strongly associated with improved OS (**Figure 7**).

**Figure 8:**
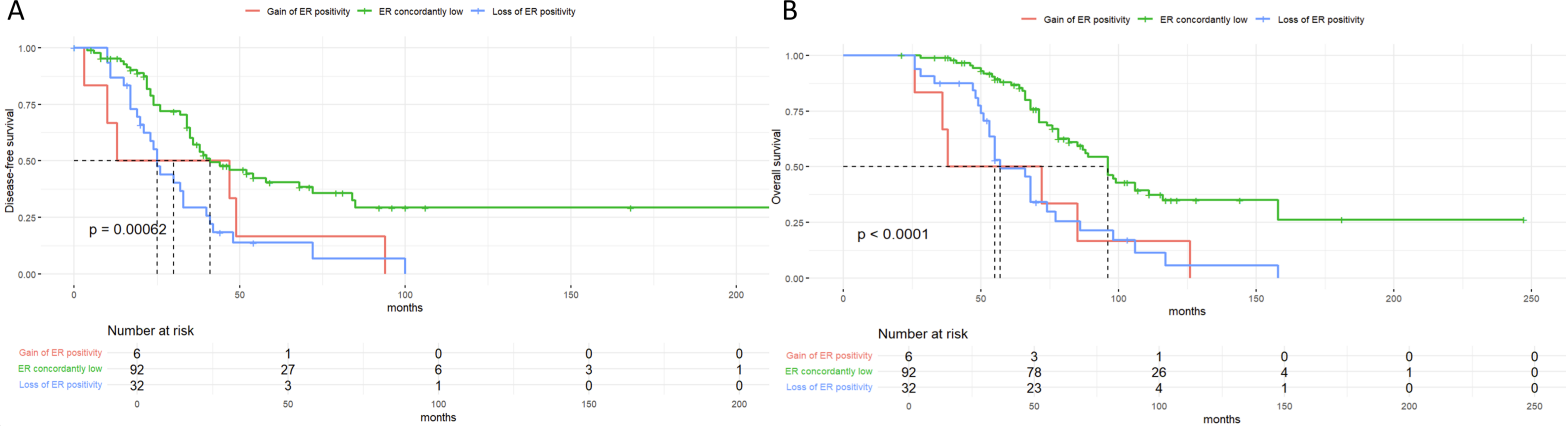
Kaplan-Meier survival curves suggest a prognostic gradient in Estrogen Receptor (ER) expression levels. **(A)** Disease-free-survival(DFS) by Estrogen Receptor (ER) status changes showing significant differences between ER subgroups (p < 0.00062). **(B)** Overall survival(OS) by Estrogen Receptor (ER) status changes showing significant differences between ER subgroups (P < 0.0001).

**Figure 9:**
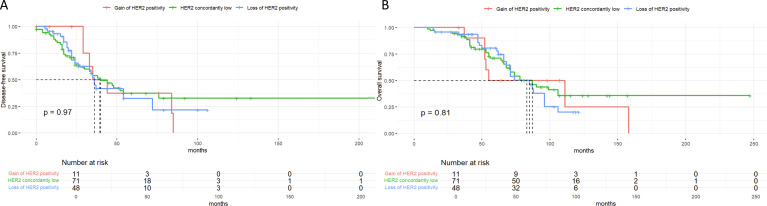
Kaplan-Meier survival curves suggest a prognostic gradient trend in HER2 expression levels. **(A)** Disease-free-survival(DFS) by HER2 status changes showing differences trend between HER2 subgroups. **(B)** Overall survival(OS) by HER2 status changes showing differences trend between HER2 subgroups.

Notably, our previous findings further support the prognostic implications of receptor expression levels. Specifically, patients with ER low expression exhibited significantly worse overall survival compared to ER-positive patients (median OS: 63.0 vs. 92.5 months, P < 0.001), yet had better outcomes than ER-negative patients (**Figure 8**). Similarly, among HER2-negative subgroups, those with HER2-low expression (IHC 1+ or 2+/ISH-) showed intermediate OS (median: 70.3 months) between HER2-zero (60.8 months) and HER2-positive patients (78.0 months) (**Figure 9**). These results suggest a prognostic gradient in both ER and HER2 expression levels, emphasizing the need for nuanced interpretation beyond binary classification.

## 4 Discussion

Intratumoral heterogeneity in breast cancer plays a crucial role in driving therapeutic resistance and facilitating metastatic progression. This study investigates key aspects, including the incidence of receptor discordance for ER, PR, and HER2 between primary and metastatic breast cancer, with ER and HER2 further stratified into zero, low, and positive expression; the determinants of receptor alterations during metastasis; and the prognostic significance of these changes on patient outcomes.

This study examined receptor profiles in 436 primary breast tumors and their matched metastatic counterparts from 363 patients. The findings revealed that ER and PR positivity rates were markedly higher in primary tumors than in metastatic lesions, whereas HER2 positivity remained relatively stable across primary and metastatic sites. The discordance rates for ER, PR, and HER2 in metastatic breast cancer were 25.1%, 33.3%, and 32.8%, respectively. The Kappa test results demonstrated strong consistency for HER2 between primary lesions and liver metastases, with ER showing good agreement overall.

A meta-analysis of receptor changes in breast cancer primary lesions and distant metastases (12)reported varying rates of receptor status changes across different studies, with discordance rates differing based on metastasis location. Specifically, the combined estimated discordance rates for ER, PR, and HER2 were 22.5% (95% CI: 16.4% to 30.0%), 49.4% (95% CI: 40.5% to 58.2%), and 21.3% (95% CI: 14.3% to 30.5%), respectively. The discordance rates for PR and HER2 observed in this study differ from the pooled estimates in the meta-analysis, which may be attributed to the significant heterogeneity among studies included in the meta-analysis and the presence of smaller sample sizes.

Compared to previous retrospective studies on receptor changes in breast cancer and metastases (6, 11), this study benefits from a larger sample size and more recent data (with metastases detected post-2013). Additionally, the study identified receptor status inconsistencies among metastases in patients who underwent multiple rebiopsies, underscoring the importance of rebiopsies to accurately confirm receptor status throughout breast cancer diagnosis and treatment. Importantly, metastatic biopsies were clinically driven rather than randomly performed. Re-biopsy was generally recommended in cases of atypical relapse patterns, early recurrence, or suspected receptor modification. This may have introduced selection bias and potentially overestimated discordance rates compared with an unselected metastatic population. Therefore, caution is warranted when generalizing these findings.

This study further investigated factors influencing receptor conversion and its prognostic relevance in metastatic breast cancer. Univariate analysis identified significant associations between receptor discordance and several factors: neoadjuvant therapy was strongly linked to ER discordance, anti-HER2 targeted therapy was significantly associated with PR and HER2 discordance, and endocrine therapy was correlated with inconsistencies across all three receptors. Lung metastasis was particularly associated with HER2 discordance. It is suggested that patients who have undergone neoadjuvant therapy, anti-HER2 targeted therapy, endocrine therapy, and lung metastasis are more recommended to undergo puncture biopsy to guide further treatment.

The mechanisms driving receptor changes in metastases are unclear but may involve intratumoral heterogeneity, clonal evolution, and treatment-induced selection pressure. Gene sequencing indicates a close genetic relationship between primary tumors and metastases, with additional mutations contributing to diversity. Whole-exome sequencing shows that untreated metastases often arise from dominant clones in primary tumors, while drug-treated metastases may come from rare clones driven by treatment pressure. This suggests that metastasis-specific mutations are more linked to drug resistance than to the metastatic process itself. Polyclonal origins are more common in untreated metastases, indicating that treatment pressure reshapes clonal evolution.

Further studies suggest that ER may reduce tumor cell invasiveness, linking receptor status changes to tumor invasion or metastasis. Whether these changes are adaptive responses during invasion and metastasis or arise from clonal selection under treatment pressure requires further investigation.

Multivariate analysis demonstrated that patients undergoing targeted therapy experienced significantly improved overall survival (OS) compared to those without such treatment. Patients with hormone receptor-positive primary tumors whose metastases also converted to hormone receptor-positive status exhibited longer OS than those with metastases remaining hormone receptor-negative. This observation is consistent with prior studies, underscoring that the prognosis of breast cancer patients with molecular alterations in metastases is predominantly determined by the receptor status of the metastases rather than the primary tumor. These findings align with Lin et al.’s work on the prognostic significance of receptor changes in bone metastases.

Unexpectedly, higher initial tumor stage was associated with a reduced risk of death in the multivariate analysis. This paradox likely reflects the composition of our metastatic cohort: patients with initial high-stage (e.g., *de novo* Stage IV) are typically treatment-naive and responsive to first-line metastatic therapy, whereas patients with initial low-stage disease who developed metastasis represent a subgroup with acquired resistance to prior adjuvant therapies, leading to poorer post-recurrence survival.

This study has certain limitations. As a retrospective, single-center analysis, it is prone to selection bias in the study population. Although guidelines recommend routine re-biopsy of metastatic lesions in metastatic breast cancer, not all patients in clinical practice undergo pathological evaluation of every metastatic site. Re-biopsy is often prioritized for patients with suspected receptor transformation based on clinical features and tumor progression, so intra-patient clustering was present in a subset undergoing multiple biopsies. Furthermore, patient slides were not uniformly re-tested or reviewed. Nevertheless, Although all metastatic lesions included in this study were evaluated by the Department of Pathology at Xi’an Jiaotong University Hospital, with receptor status of both primary and metastatic lesions reassessed in accordance with the ASCO/CAP guidelines, potential variations in measurement methods and inter-observer consistency over the 10-year period could influence the discordance rates. Finally, this study did not examine the impact of subsequent systemic treatments, leaving the question of whether receptor-based treatment adjustments in metastatic lesions improve prognosis to future research.

In summary, this study explored the heterogeneity in ER, PR, and HER2 expression between primary and paired metastatic breast cancer lesions, along with the factors influencing receptor discordance and their long-term prognostic significance. The findings suggest that neoadjuvant therapy influences changes in ER status, whereas endocrine therapy is linked to alterations in ER, PR, and HER2 expression. Additionally, targeted therapy impacts PR and HER2 expression changes. The long-term prognosis of patients is predominantly determined by the molecular subtype of metastatic lesions rather than that of the primary lesions. Approximately one-third of patients with HER2 zero expression experience a shift to HER2 low expression or HER2 amplification during metastasis. These patients may benefit from a repeat biopsy to explore treatment options with new antibody-drug conjugates (ADCs).

## Data Availability

The raw data supporting the conclusions of this article will be made available by the authors, without undue reservation.

## References

[1] Siegel RL Giaquinto AN Jemal A . Cancer statistics, 2024. CA Cancer J Clin. (2024) 74:12–49. doi: 10.3322/caac.21820. PMID: 38230766

[2] Harbeck N Penault-Llorca F Cortes J Gnant M Houssami N Poortmans P . Breast cancer. Nat Rev Dis Primers. (2019) 5:66. doi: 10.1038/s41572-019-0111-2. PMID: 31548545

[3] Pan H Gray R Braybrooke J Davies C Taylor C McGale P . 20-year risks of breast-cancer recurrence after stopping endocrine therapy at 5 years. N Engl J Med. (2017) 377:1836–46. doi: 10.1056/NEJMoa1701830. PMID: 29117498 PMC5734609

[4] Radosa JC Eaton A Stempel M Khander A Liedtke C Solomayer E-F . Evaluation of local and distant recurrence patterns in patients with triple-negative breast cancer according to age. Ann Surg Oncol. (2017) 24:698–704. doi: 10.1245/s10434-016-5631-3. PMID: 27783163 PMC5408739

[5] Kao JY Tsai JH Wu TY Wang CK Kuo YL . Receptor discordance and phenotype change in metastatic breast cancer. Asian J Surg. (2021) 44:192–8. doi: 10.1016/j.asjsur.2020.05.032. PMID: 32622530

[6] Chen R Qarmali M Siegal GP Wei S . Receptor conversion in metastatic breast cancer: analysis of 390 cases from a single institution. Mod Pathol. (2020) 33:2499–506. doi: 10.1038/s41379-020-0615-z. PMID: 32620918

[7] Grinda T Joyon N Lusque A Lefèvre S Arnould L Penault-Llorca F . Phenotypic discordance between primary and metastatic breast cancer in the large-scale real-life multicenter French ESME cohort. NPJ Breast Cancer. (2021) 7:41. doi: 10.1038/s41523-021-00252-6. PMID: 33863896 PMC8052407

[8] Walter V Fischer C Deutsch TM Ersing C Nees J Schütz F . Estrogen, progesterone, and human epidermal growth factor receptor 2 discordance between primary and metastatic breast cancer. Breast Cancer Res Treat. (2020) 183:137–44. doi: 10.1007/s10549-020-05746-8. PMID: 32613540 PMC7375990

[9] Zhao W Sun LL Dong GL Wang XR Jia Y Tong ZS . Receptor conversion impacts outcomes of different molecular subtypes of primary breast cancer. Ther Adv Med Oncol. (2021) 13:17588359211012982. doi: 10.1177/17588359211012982. PMID: 33995598 PMC8111518

[10] Lin M Jin Y Lv H Hu X Zhang J . Incidence and prognostic significance of receptor discordance between primary breast cancer and paired bone metastases. Int J Cancer. (2023) 152:1476–89. doi: 10.1002/ijc.34365. PMID: 36408915

[11] Krøigård AB Larsen MJ Thomassen M Kruse TA . Molecular concordance between primary breast cancer and matched metastases. Breast J. (2016) 22:420–30. doi: 10.1111/tbj.12596. PMID: 27089067

[12] Schrijver W Suijkerbuijk KPM van Gils CH van der Wall E Moelans CB van Diest P . Receptor conversion in distant breast cancer metastases: a systematic review and meta-analysis. J Natl Cancer Inst. (2018) 110:568–80. doi: 10.1093/jnci/djx273. PMID: 29315431

[13] Turner NH Di Leo A . HER2 discordance between primary and metastatic breast cancer: assessing the clinical impact. Cancer Treat Rev. (2013) 39:947–57. doi: 10.1016/j.ctrv.2013.05.003. PMID: 23764178

[14] Aurilio G Disalvatore D Pruneri G Bagnardi V Viale G Curigliano G . A meta-analysis of oestrogen receptor, progesterone receptor and human epidermal growth factor receptor 2 discordance between primary breast cancer and metastases. Eur J Cancer. (2014) 50:277–89. doi: 10.1016/j.ejca.2013.10.004. PMID: 24269135

[15] Duffy MJ Harbeck N Nap M Molina R Nicolini A Senkus E . Clinical use of biomarkers in breast cancer: updated guidelines from the European Group on Tumor Markers (EGTM). Eur J Cancer. (2017) 75:284–98. doi: 10.1016/j.ejca.2017.01.017. PMID: 28259011

[16] Cardoso F Senkus E Costa A Papadopoulos E Aapro M André F . 4th ESO-ESMO international consensus guidelines for advanced breast cancer (ABC 4)†. Ann Oncol. (2018) 29:1634–57. doi: 10.1093/annonc/mdy192. PMID: 30032243 PMC7360146

[17] Rugo HS Rumble RB Macrae E Barton DL Connolly HK Dickler MN . Endocrine therapy for hormone receptor-positive metastatic breast cancer: American Society of Clinical Oncology guideline. J Clin Oncol. (2016) 34:3069–103. doi: 10.1200/jco.2016.67.1487. PMID: 27217461

[18] Curigliano G Burstein HJ Winer EP Gnant M Dubsky P Loibl S . De-escalating and escalating treatments for early-stage breast cancer: the St. Gallen International Expert Consensus Conference on the Primary Therapy of Early Breast Cancer 2017. Ann Oncol. (2017) 28:1700–12. doi: 10.1093/annonc/mdx308. PMID: 28838210 PMC6246241

[19] Wolff AC Somerfield MR Dowsett M Hammond MEH Hayes DF McShane LM . Human epidermal growth factor receptor 2 testing in breast cancer: ASCO-College of American Pathologists guideline update. J Clin Oncol. (2023) 41:3867–72. doi: 10.1200/jco.22.02864. PMID: 37284804

[20] Allison KH Hammond MEH Dowsett M McKernin SE Carey LA Fitzgibbons PL . Estrogen and progesterone receptor testing in breast cancer: ASCO/CAP guideline update. J Clin Oncol. (2020) 38:1346–66. doi: 10.1200/jco.19.02309. PMID: 31928404

