# Supplementary material for: A decade of receptor discordance and phenotypic transitions in metastatic breast cancer: a single-center retrospective study of 363 cases
Source: Front Oncol. 2026 Apr 20;16:1797175. doi: 10.3389/fonc.2026.1797175 (PMC13135980; doi:10.3389/fonc.2026.1797175)
Supplement: Supplementary file 1 [file Table1.docx]

**TableS1**. Inconsistency in receptor status among different metastatic lesions

| Metastatic sites | ER | | | PR | | | HER2 | | |
| --- | --- | --- | --- | --- | --- | --- | --- | --- | --- |
|  | Concordant | Discordant | Rates(%) | Concordant | Discordant | Rates(%) | Concordant | Discordant | Rates(%) |
| Liver(96) | 72 | 24 | 25.0% | 58 | 38 | 39.5% | 39 | 57 | 59.3% |
| Lung(70) | 48 | 22 | 31.4% | 44 | 26 | 37.1% | 16 | 54 | 77.1% |
| Bone(29) | 20 | 9 | 31.0% | 19 | 10 | 34.4% | 11 | 18 | 62.0% |
| Chest wall(81) | 64 | 17 | 20.9% | 59 | 22 | 27.1% | 36 | 45 | 55.5% |
| Pleura(19) | 11 | 8 | 42.1% | 10 | 9 | 47.3% | 8 | 11 | 57.8% |
| Lymph nodes(107) | 84 | 23 | 21.4% | 73 | 34 | 31.7% | 41 | 66 | 61.6% |
| Skin(10) | 8 | 2 | 20.0% | 9 | 1 | 10.0% | 2 | 8 | 80.0% |
| Brain(5) | 4 | 1 | 20.0% | 4 | 1 | 20.0% | 1 | 4 | 80.0% |
